# Supplementary material for: Community perceptions of vaccination among influential stakeholders: qualitative research in rural India
Source: BMC Public Health. 2021 Nov 18;21:2122. doi: 10.1186/s12889-021-12188-4 (PMC8600485; doi:10.1186/s12889-021-12188-4)
Supplement: Supplementary file 1 — Additional file 1: Supplementary File 1. Supplementary File_Focus Group guides_24 Sept 2019_Clean. “Community Perceptions on Immunizations: Focus Group Discussions”. These guides were designed based on our research questions and our objectives, and they aimed to facilitate the flow of the focus group discussions among the target groups. [file 12889_2021_12188_MOESM1_ESM.docx]

**Community Perceptions on Immunizations: Focus Group Discussions**

**FOCUS GROUP GUIDE FOR TARGET GROUPS**

**Group I. Focus Group guide for New Expectant Mothers**

1. What have any of you heard about vaccinations?
   1. ***Probe***: positive or negative information? Personal stories?
   2. ***Follow-up:*** Who did you hear this from? ***Probe:*** family; health workers; media; other mothers
2. Have any of you started thinking about vaccinating your child?
   1. ***Follow-up:*** Are there challenges you may face when you take your child to the immunization clinic to get vaccinated?
   2. ***Follow up:*** What is making you think about vaccinations? (probe: family, healthcare worker, media)
3. Who would you all trust to provide you with vaccination information?
   1. ***Probe***: mothers; family; doctor; media; neighbors; Anganwadi workers, government employees; community members
4. What other benefits (other than vaccines) do any of you think you can get from visiting the immunization clinic or the nurse who gives vaccines?
   1. ***Probe*:** child care, sick child care, child feeding practices, hygiene (such as washing hands), child safety, and education for your child, nutritional supplements, and social interaction benefits
5. Have any of you observed any child get harmed or have you had any bad experiences with vaccinations?
6. Who in your respective homes will likely be involved with making decisions about your child’s vaccinations?
   1. ***Probe:*** influence of father, mother, mother-in-law

**Group II. Focus Group guide for Fathers of Children under 5**

1. Are immunizations discussed in any of your homes?
   1. ***Probe***: with who? **When** did you start discussing? When your wife was pregnant? After birth? During the first well-visit?
2. Who in your homes typically takes children to the immunization clinic?
   1. ***Probe***: Who is present? Do you (the father) ever attend – why or why not?
3. Who do you all trust to provide you with vaccination information?
   1. ***Probe***: family; doctor; media; neighbors; Anganwadi workers, government employees; community members
4. Have any of you observed any child get harmed or have any bad experiences from vaccinations?
5. What other benefits (other than vaccines) have any of your children received from visiting the immunization clinic?
   1. ***Probe*:** child care, sick child care, child feeding practices, hygiene (such as washing hands), child safety, and education for your child, nutritional supplements, and social interaction benefits
   2. ***Follow-up:*** What other benefits (other than vaccines) would you want to receive from visiting the immunization clinic?
6. What experiences have influenced any of your decisions regarding vaccinations?
   1. ***Probe***: interactions with religious leaders; doctors; health personnel; teachers
   2. ***Probe***: government campaigns
7. How would all of you describe your conversations about vaccines with other fathers in the community?
   1. ***Probe***: Have these influenced your decisions to vaccinate?

**Group III. Focus Group guide for Anganwadi workers and ASHA workers**

1. What motivates you all to discuss vaccinations with community members?
2. Do any of you face challenges when visiting families and asking them to get their children vaccinated?
   1. ***Probe:*** disinterested; forget information; fearful of vaccines; misinformed
   2. ***Follow-up:*** Can you describe any particular interactions that you remember?
   3. Probe: Challenges families face; taking day off work; having someone watch their other kids; cost implications; distance to center.
3. What challenges do any of you notice in families bringing their children to get vaccinated?
   1. ***Probe:*** Are families giving anything up by bringing their children to immunization clinics? Take day off from work; have to find someone to watch other kids
4. In your experiences, why do some families choose not to vaccinate their children?
5. What strategies have you all use when talking with parents about vaccinations and having them come to the vaccine clinics?
   1. ***Follow-up:*** Which have been useful? Why?
6. What do any of you think families consider to be the benefits of visiting the immunization clinic?
   1. ***Probe:*** receive other information; meet other mothers; space for conversation

**Group IV. Focus Group guide for Community Influencers**

1. What are the biggest priorities you all have in managing community affairs as leaders?
   1. ***Probe:*** education; health; vaccinations; maternal mortality; nutrition
2. Do you feel that your communities have a good understanding of what vaccines do?
3. Who do your communities trust to provide them with vaccination information?
   1. ***Probe***: family; doctor; media; neighbors; Anganwadi workers, government employees; other community members
4. Who plays a role in decision making regarding children’s vaccinations in your respective communities?
5. How do you all see your role in families’ decisions about vaccinating their children?
   1. ***Follow-up:*** Do you see your role as being influential?
6. What do you all think are the benefits of having a child go to the clinic ?
   1. ***Probe:*** Are there benefits other than vaccines?
   2. ***Follow-up:*** How do you think the family benefits?
7. Do any of you feel that the people find it hard to go to the clinic or to the Anganwadi worker to have their child vaccinated?
   1. ***Follow-up:*** What are their challenges?
8. Do you think that every child in your respective communities should be vaccinated? Why or why not?
